# Classification of Documented Goals of Care Among Hospitalized Patients with High Mortality Risk: a Mixed-Methods Feasibility Study

**DOI:** 10.1007/s11606-024-08773-z

**Published:** 2024-05-06

**Authors:** Catherine L. Auriemma, Anne Song, Lake Walsh, Jason J. Han, Sophia R. Yapalater, Alexander Bain, Lindsay Haines, Stefania Scott, Casey Whitman, Stephanie P. Taylor, Scott D. Halpern, Katherine R. Courtright

**Affiliations:** 1 Palliative and Advanced Illness Research (PAIR) Center, University of Pennsylvania, Philadelphia, PA USA; 2 Department of Medicine, University of Pennsylvania, Philadelphia, PA USA; 3 Leonard Davis Institute of Health Economics, University of Pennsylvania, Philadelphia, PA USA; 4 Division of Internal Medicine and Primary Care, Brigham and Women’s Hospital, Boston, MA USA; 5 Department of Surgery, University of Pennsylvania, Philadelphia, PA USA; 6 Division of Pulmonary and Critical Care, New York University-Langone, New York, NY USA; 7 Division of Hospital Medicine, University of Michigan, Ann Arbor, MI USA

## Abstract

**Background:**

The ability to classify patients’ goals of care (GOC) from clinical documentation would facilitate serious illness communication quality improvement efforts and pragmatic measurement of goal-concordant care. Feasibility of this approach remains unknown.

**Objective:**

To evaluate the feasibility of classifying patients’ GOC from clinical documentation in the electronic health record (EHR), describe the frequency and patterns of changes in patients’ goals over time, and identify barriers to reliable goal classification.

**Design:**

Retrospective, mixed-methods chart review study.

**Participants:**

Adults with high (50–74%) and very high (≥ 75%) 6-month mortality risk admitted to three urban hospitals.

**Main Measures:**

Two physician coders independently reviewed EHR notes from 6 months before through 6 months after admission to identify documented GOC discussions and classify GOC. GOC were classified into one of four prespecified categories: (1) comfort-focused, (2) maintain or improve function, (3) life extension, or (4) unclear. Coder interrater reliability was assessed using kappa statistics. Barriers to classifying GOC were assessed using qualitative content analysis.

**Key Results:**

Among 85 of 109 (78%) patients, 338 GOC discussions were documented. Inter-rater reliability was substantial (75% interrater agreement; Cohen’s kappa = 0.67; 95% CI, 0.60–0.73). Patients’ initial documented goal was most frequently “life extension” (*N* = 37, 44%), followed by “maintain or improve function” (*N* = 28, 33%), “unclear” (*N* = 17, 20%), and “comfort-focused” (*N* = 3, 4%). Among the 66 patients whose goals’ classification changed over time, most changed to “comfort-focused” goals (*N* = 49, 74%). Primary reasons for unclear goals were the observation of concurrently held or conditional goals, patient and family uncertainty, and limited documentation.

**Conclusions:**

Clinical notes in the EHR can be used to reliably classify patients’ GOC into discrete, clinically germane categories. This work motivates future research to use natural language models to promote scalability of the approach in clinical care and serious illness research.

**Supplementary Information:**

The online version contains supplementary material available at 10.1007/s11606-024-08773-z.

## INTRODUCTION

Delivery of goal-concordant care—that is, medical care that aligns with and promotes patients’ goals and preferences regarding treatment intensity, functional outcomes, and longevity—is widely supported as the highest quality of care.^1,2^ Indeed, goal-concordant care has been identified as a key priority by the National Academy of Medicine and rated by an expert panel as the most important outcome measure for studies of advance care planning.^3,4^ In order to provide goal-concordant care, clinicians must engage patients or their surrogate decision-makers in goals-of-care (GOC) discussions to elicit their values, goals, and care preferences,^5,6^ and then document those conversations in the electronic health record (EHR) to optimally guide the future care they receive.^1,7,8^

Recent advances in natural language processing and machine learning methods have been applied to identify documented GOC discussions from clinical notes in the EHR to inform quality improvement initiatives and evaluate palliative care interventions.^9–11^ However, distinguishing among different goals within documented GOC discussions has proved a more challenging task.^12^ Prior studies have relied on more readily available, limited proxies for GOC such as code status or physician orders for life-sustaining treatments because they are more readily available,^13–16^ or focused on a disease-specific treatment preference thus limiting generalizability.^17,18^ Other studies have queried patients directly about their GOC,^9,19,20^ an approach that suffers from data missingness, failure to capture the dynamic nature of patients’ goals, and impracticality for large clinical trials. These limitations could potentially be mitigated by using clinical notes to classify patients’ GOC, but the feasibility and reliability of this approach is unknown.

Several frameworks exist for classifying GOC to enable the measurement of goal-concordant care.^21–23^ One framework for measuring goal-concordant care by co-author Halpern proposes using clinical notes in the EHR to classify GOC, which, if feasible, would enable a generalizable and pragmatic method that can be broadly applied in clinical research and clinical care. We thus performed a retrospective mixed-methods study to evaluate the feasibility of classifying patients’ GOC from clinical EHR notes among a cohort of patients hospitalized with serious illness. We also describe the frequency and patterns of changes in patients’ goals over time and identify barriers to reliable goal classification. Limited results from this study were reported in abstract.^24,25^

## METHODS

### Design

We conducted a retrospective chart review study among 120 randomly selected patients hospitalized between April 1, 2019, and July 31, 2019, at the University of Pennsylvania Health System (UPHS). We employed an explanatory sequential (quantitative to qualitative) mixed-methods approach^26^ to identify possible mechanisms for challenges encountered in classifying goals and potential opportunities to refine the GOC classification framework. This study was approved with a waiver of informed consent by the Institution Review Board at the University of Pennsylvania.

### Study Population and Setting

For this feasibility, mixed-methods study of a novel GOC classification method, we targeted an analytic sample size of 100 unique patient encounters. Eligible patient encounters included adults (≥ 18 years of age) admitted for ≥ 3 calendar days to one of three urban, academic-affiliated hospitals at UPHS. To achieve our primary study objective, we sought a patient sample enriched for documented GOC discussions while also avoiding introducing selection bias (as would occur with purposive sampling of charts based on the presence of a documented GOC discussion). Thus, we first restricted the eligible sample to patients with ≥ 50% risk of death within 6 months of admission, for whom there is expert consensus that a GOC discussion is recommended prior to hospital discharge.^27^ Six-month mortality risk was determined by an EHR-based mortality prediction model previously developed and validated in the study hospitals.^28^ To enable our secondary longitudinal assessment of patients’ GOC, patients must also have had at least one prior inpatient or outpatient encounter at UPHS within 12 months prior to the eligible admission. We then employed random sampling stratified evenly across high vs very high 6-month mortality risk (50–74% high and ≥ 75% very high) to identify 120 patient charts. After removing one duplicate patient chart, we piloted and refined the classification framework on 10 charts, with the remaining to be used as the final analytic sample.

### Data Collection and Variable Definitions

#### Patient Characteristics

Patients’ self-reported sociodemographic data were obtained from the UPHS EHR clinical data warehouse: binary sex, race, ethnicity, religion, and primary language. Race, ethnicity, and primary language were analyzed as binary variables due to low numbers of subjects in minority categories. An inpatient palliative care consultation was identified by a signed consult order during the index hospital encounter. During the 6-month follow-up period, we collected all hospital readmissions, home care visits, and subsequent palliative care consultations.

#### Goals of Care

A GOC discussion was defined as a conversation between a clinician and a patient and/or patient’s surrogate decision-maker about the patient’s values or goals with respect to their preferences for medical care that was documented anywhere in an EHR clinical note.^5,19^ Isolated mentions of code status without an accompanying discussion of broader goals were not included.

The study lead investigators (CA, AS, KC) applied an adapted framework for classifying goals of care from EHR data into five initially prespecified categories: “comfort-focused care,” “maintain or improve function,” “life extension,” “unclear,” and “making it to a life event.”^5,21,29^ We conducted initial consensus chart reviews on 10 randomly selected charts (5 high-risk mortality and 5 very-high-risk mortality) to identify GOC conversations and to refine GOC categories and definitions. Charts were reviewed both independently and as a team to build consensus through group discussion. This process resulted in a retention of four categories of GOC (Table 1), with a consolidation of “making it to a life event” into the broader category of “life extension” due to its very low prevalence. These charts were excluded from the final analytic sample and reserved for coder training on GOC identification and categorization.

**Table 1:** Goals of Care Classification, Definitions, and Representative Text from the Electronic Health Record

Category	Definition	Representative text
Comfort-focused	Patient’s goal is to maximize comfort and avoid suffering. Includes seeking interventions to promote comfort (e.g., pain control) and avoiding interventions that would increase discomfort, even at the expense of decreasing longevity	The patient is deciding to go home on hospice. In the event her heart were to stop, she does not want CPR. She additionally does not want to be intubated, placed on a ventilator or transferred to the MICU. Her wish is to go home
		The family was updated on the patient’s acute decompensation this morning…I explained what would happen her heart were to fail and we would need to code her. Both her sister and son expressed that they would want her to be comfortable and would not want her to go through the trauma of the code. At the bedside, we told the patient that her body is dying and we are reaching the ceiling of our life support medications. The patient said she feels God is calling her home. She affirmed that she does not want to be coded or intubated and gave permission to stop dialysis…She also requested to speak with a Chaplain. She expressed fear of death and pain and accepted pain medications
		The patient stated she did not want to continue and interested in transitioning to comfort care…I spoke with [the patient’s daughter] who was present at bedside today…I expressed what the patient communicated re: her own GOC to [daughter]. [Daughter] was emotionally upset about the discussion of end-of-life…She felt that removing life support is ‘murder or passive suicide.’ …I repeatedly expressed that no decisions need to be made but rather this was an opportunity to talk with everyone, including the patient who can express her own wishes. Throughout this conversation, the patient was nodding yes and confirming what she had expressed the other day re: her own GOC. She was able to mouth words to her daughter that she say she’s ready to transition
		Goals are palliative, plan for transition to in-patient hospice. Wife hoping to best honor patient’s wishes and provide a death that is dignified
Maintain or improve function	Patient’s goal is to maintain or improve cognitive or physical functioning by undergoing medical care aimed at preventing or reversing dysfunction, even if that medical care would increase discomfort. However, care that would increase survival/longevity without preservation or improvement in function is generally avoided	Extensive discussion of the options, pros and cons of more aggressive therapy being the most important. Will continue to hold off any chemotherapy for now—his quality of life is getting slowly better and disease is not aggressive. In terms of goals of therapy, the most important would be a return to his level of functioning of two years ago
		Patient and I discussed her short-term goals of care. She says she would ideally like to return to work where she is a manager at an adult daycare center. She says she finds this work meaningful
		Patient’s wife said she is processing patient’s relatively new diagnosis and rapid decline. Patient had previously expressed to wife that he values quality of life, meaning functional independence. He does not want to suffer. Patient’s wife trying to balance honoring patient’s wishes with gathering all of the date from the team to support making decisions on his behalf. Wife also said patient would not want to be intubated or have CPR given the state of his current illness…Patient has previously expressed to [wife] that he wants to be functionally well and independent and values quality of life
		Patient understands that she can stop [chemotherapy] at any time. Goal is to help her feel better. If makes her feel worse, recommendation at that time would be for hospice. Patient is DNR/DNI and I signed out of hospital DNR form for her
Life extension	Patient’s goal is to live as long as possible without limitations on care. Extending longevity or survival is prioritized over maximizing function or comfort	[Patient], [spouse] and I discussed what [patient]’s preference would be in the event of a life-threatening emergency or cardiac arrest. She tells me that she would like a ‘second chance’ at all costs and would want her medical team to exhaust all possible options, including performing resuscitation in the event of cardiac arrest
		We reviewed the options available to her regarding treatment of her lymphoma. She continues to want to pursue continued therapy, even if there is only a small chance of a temporary remission. [Oncologist] explained that if she were to improve enough from a pulmonary standpoint she would potentially be able to receive more therapy. I tried to elicit her values, and she tearfully expressed only a desire not to die. She expressed that her goal would be to be able to visit the beach and stand in the sand again
		‘I just want to get better.’ Daughter is concerned about improving her appetite so she can gain some weight…I want to spend the rest of my life with my children, grandchildren, and great grandchildren. My Longevity…I can probably adapt to anything….Anything necessary to prolong life including life support and ICU care. ‘Whatever is necessary, there is nothing I don’t want.’
		Husband was updated. I also shared that I had not seen any significant neurologic recovery. He said ‘just do whatever you can to keep her alive’; not interested in discussing goals of care further

**Figure 1:**
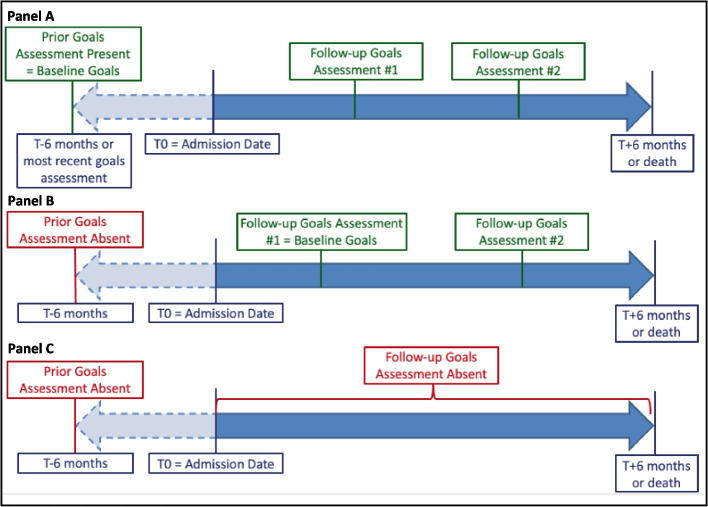
Study follow-up and identification of goals-of-care (GOC) assessments over time based on presence or absence of GOC assessments at different time points. **Panel A** Patient record with documented GOC discussions in the 6 months prior to enrollment date and during follow-up. **Panel B** Patient record with no documented GOC in the 6 months prior to enrollment date but multiple GOC assessments during follow-up. **Panel C** Patient record with no documented GOC in the 6 months prior to enrollment date or during follow-up.

GOC identification and categorization was then completed by six trained coders, all of whom were physicians-in-training (two fellows (CA, critical care; LH, palliative care) and four residents (AS, SY, AB; internal medicine; JH, cardiac surgery). Two coders independently reviewed all EHR notes spanning inpatient, ambulatory, and home care visits from 6 months prior to the index admission date through 6 months after and identified (1) a baseline GOC discussion, defined as the one preceding but most proximate to the index admission date or, if none, the first discussion after the index admission date, and (2) all subsequent GOC discussions from admission through death or 6 months (Fig. 1). We also recorded the EHR note type (e.g., history and physical, progress, advance care planning, consult note) in which the GOC discussion was documented. Disagreements in whether a note excerpt comprised a GOC discussion were resolved by consensus between coders.

Once all GOC discussions were identified in the patient cohort, pairs of coders independently applied the adapted GOC framework to classify goals identified within each documented discussion into one of the four categories. Coders were blinded to each other’s classifications and coder pairs varied such that multiple combinations of coders were used, but all charts were reviewed by one of the fellows (CA, LH). GOC discussions for which there was true disagreement in goals classification between coders (i.e., not due to coder error) and those for which goals were classified as “unclear” were reviewed and adjudicated by two physicians with expertise in critical care and palliative care (CA (when not part of the coding pair) and KC).

### Analyses

#### Statistical Analyses

We calculated the raw agreement percentage between coders for GOC classification and measured interrater reliability using Cohen’s kappa statistic. The primary unit of analysis was a GOC discussion. In secondary analyses, we explored patient-level changes in GOC over time and baseline characteristics associated with GOC documentation and a change in GOC in univariate analyses using Student’s *t* test, Pearson’s chi-square test, or Mann–Whitney-Wilcoxon test as appropriate. Statistical analyses were conducted using Stata v17 (College Station, TX), and significance was set at *p* < 0.05 for 2-tailed tests.

#### Qualitative Assessment

To identify sources of coder disagreement and ambiguity with the classification schema, we qualitatively assessed all GOC discussions for which (1) coders disagreed on the goal classification, or (2) the goal was classified as “unclear.” We systematically identified major themes from those data in three stages.^30^ First, one coder who was not involved in the initial GOC coding (LW) reviewed the foregoing GOC discussions using a process of open coding that included generating a list of codes as they emerged inductively from the data. All codes were then discussed by the study team, refined, and defined. Second, two coders (LW and AS) independently reviewed the GOC discussions and assigned applicable codes. Disagreements between coders were resolved by consensus. Finally, the team identified patterns across the codes and combined them into major themes.

## RESULTS

### Patient Cohort Characteristics

The cohort consisted of 109 unique patients (Supplemental Table 1) with a median age of 70 years (interquartile range (IQR) 63, 79). Fifty-three (49%) patients were female. Sixty-three patients (58%) identified as white and forty-one patients (38%) identified as Black. Most patients (*n* = 83, 76%) were insured by Medicare. Fifty patients (45%) had a diagnosis of metastatic cancer. The median Elixhauser comorbidity index was 6 (IQR 4,8), and the median 6-month mortality risk was 62% (IQR 55%, 66%) in the high-risk stratum, and 81% (IQR 78%, 87%) in the very-high-risk stratum. Eighty-five (78%) patients were admitted from the emergency department and 35 (32%) spent time in the ICU during the index hospitalization. Forty-three (39%) patients received a palliative care consultation during the index hospitalization. The median length of stay for the index hospitalization was 7.8 days (IQR 4.8, 12.0) and 16 (15%) patients died in the hospital. Among the 93 patients who survived the index hospitalization, 14 (15%) had a follow-up palliative care consultation after discharge, 61 (66%) were enrolled in home care, 54 (58%) had ≥ 1 readmission within 6 months, and 34 (37%) died during follow-up.

### Documented Goals-of-Care Discussions

**Figure 2:**
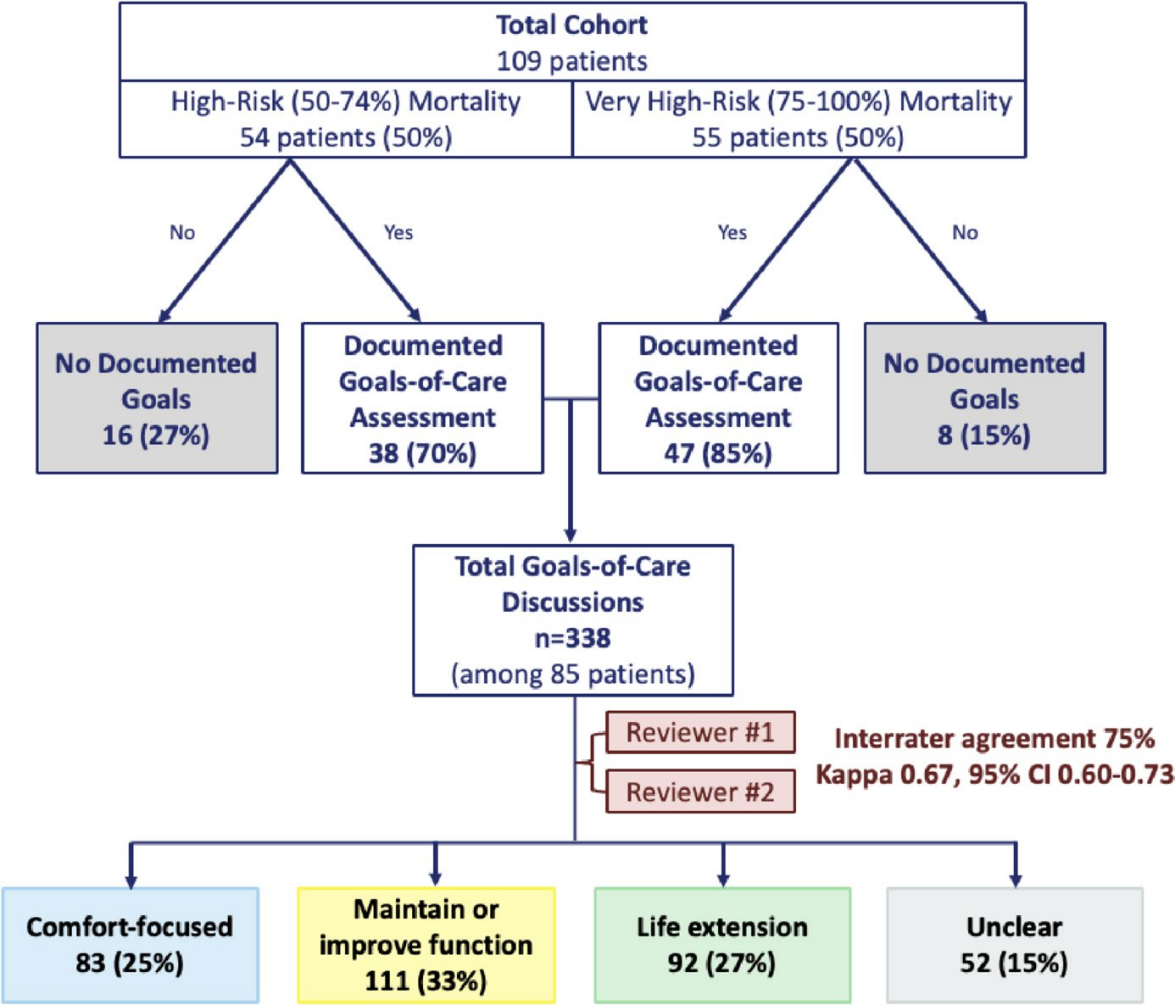
Study flow and goals-of-care discussion classification.

A total of 338 documented GOC discussions were identified among 85 of 109 (78%) patients (Fig. 2). Seventy-seven (71%) patients had > 1 documented GOC discussion during the study period (median 3 GOC discussions per patient [IQR 1, 5]). A baseline GOC discussion was documented in the 6 months prior to the index hospitalization for 49 of the 85 patients (45%). GOC discussions were documented within inpatient, ambulatory, and home care encounters and most commonly in advance care planning (ACP) notes (35%), followed by inpatient progress notes (25%) and inpatient consult notes (14%) (Supplemental Table 2). Among all 109 patients, those who died during the study period had more GOC discussions compared to those who survived (median 4 [IQR 3, 6] vs 1 [IQR 0, 3], *p* =  < 0.001).

### Categorization of Goals

Inter-rater reliability between coders for classifying GOC was substantial (75% inter-rater agreement; Cohen’s kappa = 0.67; 95% CI, 0.60–0.73). Inter-rater agreement was highest for goals classified as “comfort-focused” (90%) compared to “life extension” (74%), “maintain or improve function” (72%), and “unclear” (68%). Among the 57 goals initially classified as “unclear” by both coders, five (9%) were reclassified in the adjudication process. Overall, the most common GOC was “maintain or improve function” (*N* = 111, 33%), followed by “life extension” (*N* = 92, 27%), “comfort-focused” (*N* = 83, 25%), and “unclear” (*N* = 52, 15%) (Fig. 2). Excerpts of clinical text from GOC discussions representative of each goal type are provided in Table 1.

### Trajectory of Goals of Care over Time

**Figure 3:**
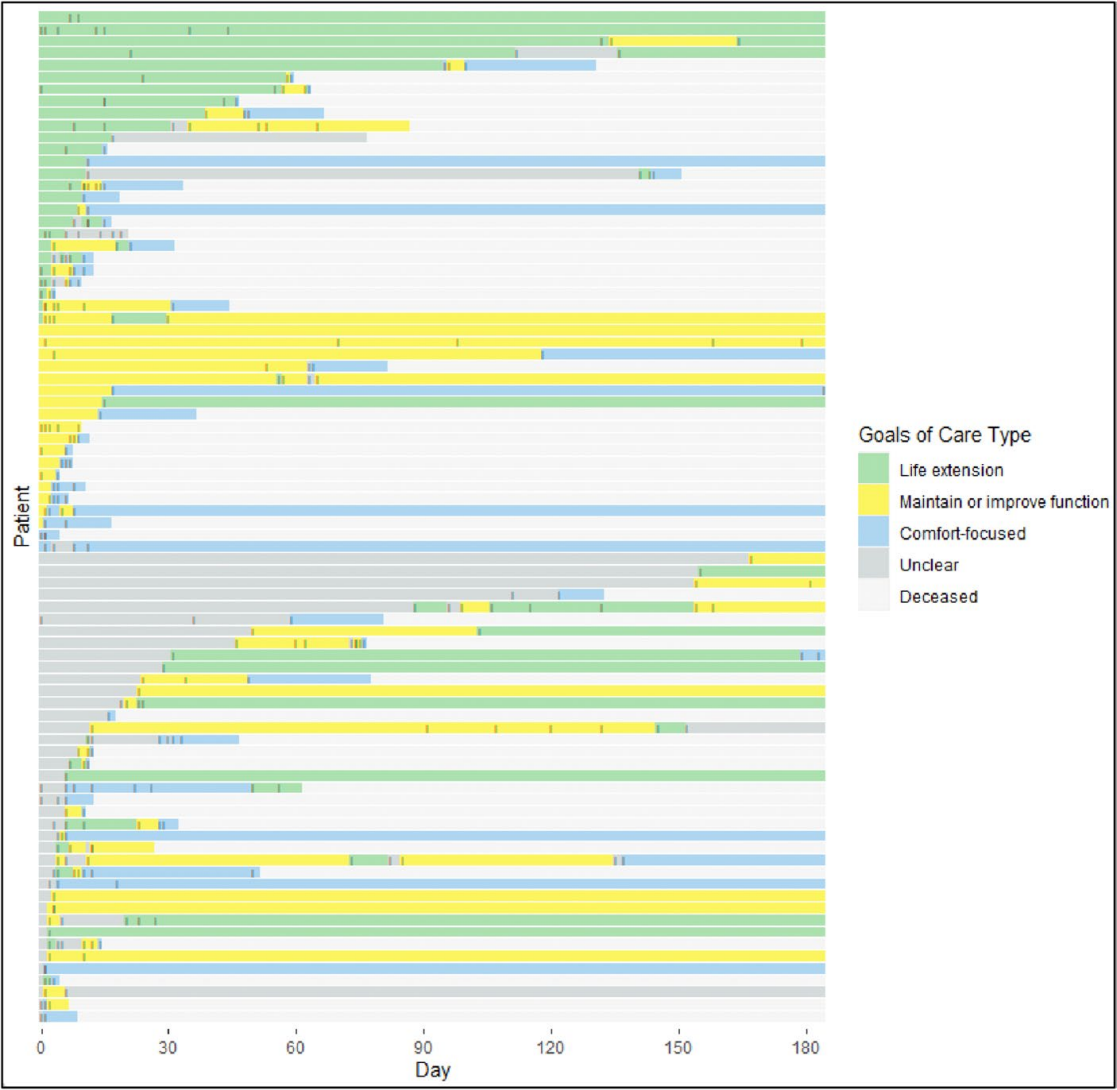
Tile plot showing frequency and classification of patients’ goals-of-care discussions over time. Made with R Core Team (2021). Ggplot. https://www.R-project.org/. Each patient is represented on the y-axis. Gray dashes denote individual goals-of-care discussions for each patient during the study period.

**Figure 4:**
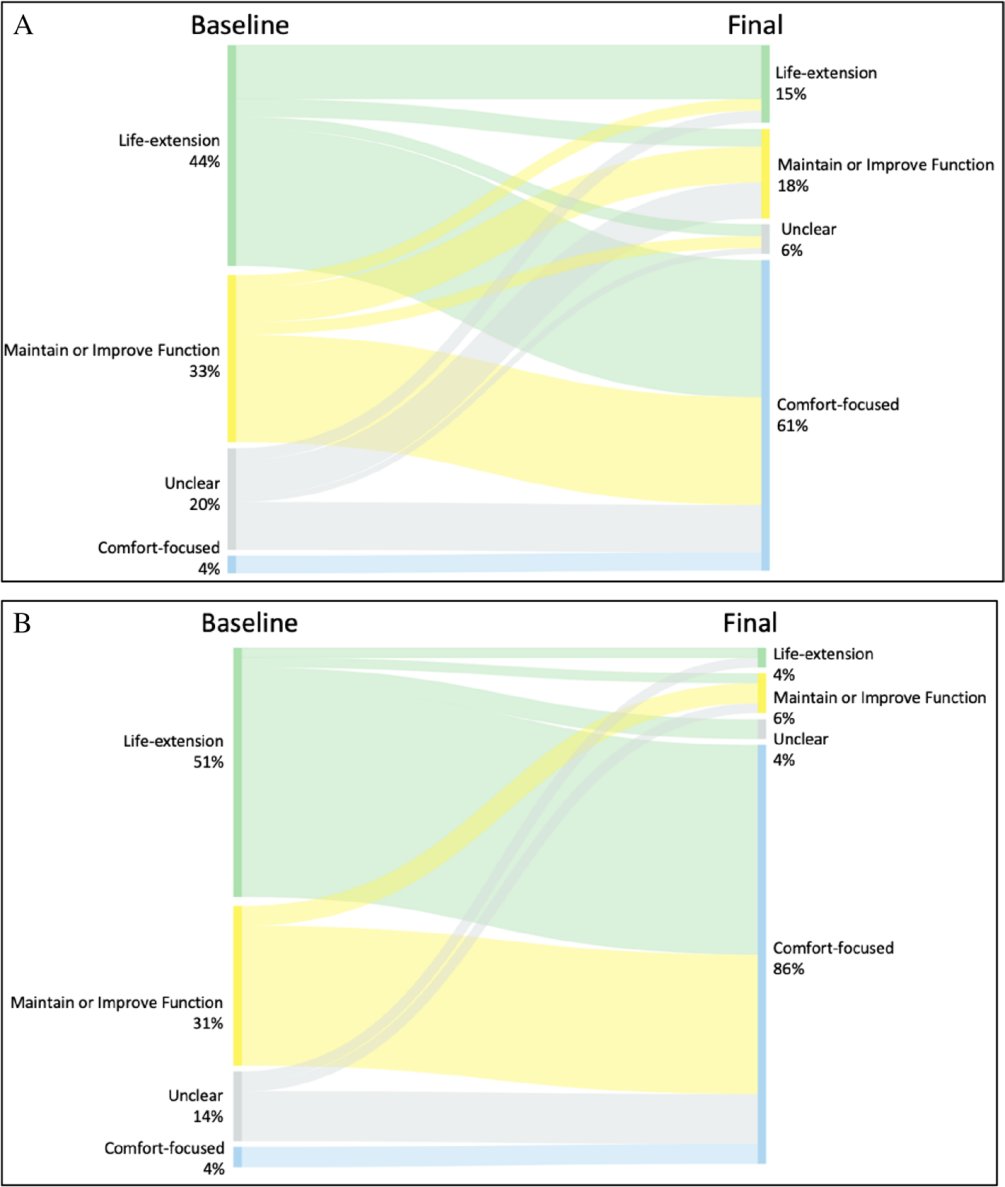
Alluvial flow diagrams illustrating changing distribution of goals of care (GOC) from baseline to final. **Panel A** All patients with ≥ 1 GOC discussion (*N* = 85). Last observation carried forward for 8 patients with only 1 GOC discussion. **Panel B** Decedent patients with ≥ 1 GOC discussion (*N* = 49). Last observation carried forward for 1 patient with only 1 GOC discussion. Made with SankeyMATIC (https://sankeymatic.com/build/).

Among the 85 patients with at least one GOC discussion, the most common GOC at baseline was “life extension” (*N* = 37, 44%), followed by “maintain or improve function” (*N* = 28, 33%), “unclear” (*N* = 17, 20%), and “comfort-focused” (*N* = 3, 4%). Baseline GOC differed by patient sex (female: 29% life extension, 39% function, 7% comfort, 24% unclear vs male: 57% life extension, 27% function, 0% comfort, 16% unclear; *p* = 0 0.03)) and baseline predicted mortality (high risk: 34% life extension, 34% function, 0% comfort, 32% unclear vs very high risk: 51% life extension, 32% function, 6% comfort, 11% unclear; *p* = 0.04). Among the 77 (71%) patients with more than one GOC discussion, 66 (86%) had a different subsequent goal, which was most commonly comfort-focused (*N* = 49, 74%) (Fig. 3). Of the 17 (20%) patients whose initial goals were classified as *unclear*, 16 (94%) subsequently had a documented GOC discussion from which the goals were able to be classified into one of the other three categories (Fig. 4A). The only patient-level characteristic associated with an increased likelihood of changing GOC was having a diagnosis of metastatic cancer (100% vs 34%, *p* = 0.01). Among decedents with more than one documented GOC discussion (*N* = 49), 45 (92%) changed goals during the study period, with the majority (*N* = 42, 85%) expressing “comfort-focused” goals prior to death (Fig. 4B). The last documented GOC discussion occurred a median of 2.6 days prior to death (0.4, 10.5).

### Qualitative Analysis of Coder Disagreement and “Unclear” Goals

Of the 79 GOC discussions with initial inter-rater differences, 58 (73%) GOC discussions represented true coder disagreement requiring adjudication and were included in the qualitative assessment, along with the 52 (15%) GOC discussions classified as “unclear.” For the total 110 GOC discussions qualitatively reviewed, interrater agreement and reliability between coders were nearly perfect (95% interrater agreement; Cohen’s kappa = 0.93; 95% CI, 0.80–1.0). We identified three major themes: (1) multiple goals concurrently held or conditional; (2) explicit or implicit uncertainty; and (3) insufficient documentation (Table 2). Insufficient documentation was the most common theme among the 52 GOC discussions for which goals were classified as “unclear” (23, 44%), whereas multiple goals concurrently held or conditional was the most common theme among the 58 GOC discussions with true inter-rater disagreement (31, 53%).

**Table 2:** Themes and Subthemes Identified from Qualitative Review of Goals-of-Care Discussions with Inter-Rater Disagreement (*N* = 58) or Unclear Goal Classification (*N* = 52)

Theme and definition	Representative text
**Multiple goals concurrently held or conditional** Multiple goals are held concurrently that cross distinctions between the three categories; or the stated goals are linked to some conditional health state or circumstance	Patient tells me he feels very unwell on dialysis. But also that he wants to live. At this time he does not want dialysis, not today
	Patient would like to start TPN with goal of moving toward chemotherapy. Patient and I have had some conversations about her being in control of when to stop TPN, if side effects, limitations on mobility, etc. are intolerable to her…Patient says that she is moving forward with the decision for TPN with the hope that she will ultimately be able to receive chemotherapy. She reflected that she does not have answers about the microperforations, and will have to wait and see how these develop and how her body responds. Yesterday we had discussed that she might not want TPN if these things were not treatable, and we briefly reflect on that conversation by saying that if she ever wants to stop TPN, she is able to make that decision…Pain is tolerable today, and well balanced with mental clarity, per patient
**Explicit or implicit uncertainty** ** Subtheme from GOC discussions with unclear goals**: patient/family explicitly expresses uncertainty as they await more information, more time, or an additional person’s input ** Subtheme from GOC discussions with inter-rater disagreement**: patient/family express goals implicitly and require inference	Dr. and team called by this afternoon to discuss surgical options, expectations etc. Patient is with her husband and other family members in room. Wants some time to decide about her ultimate goals and how aggressive she wants to be with regard to palliation
	Held family meeting with patient’s children, grandchildren, nieces and nephews via conference call. Patient poor prognosis was discussed, as well as limited options for treatment. Family would like time (1–2 days) to discuss among themselves whether they would like to continue aggressive medical management or move to more comfort-oriented measures
	Long discussion today with [the patient] and her daughter about continued progression of her cancer, causing significant morbidity and requiring multiple hospitalizations. We recommend best supportive care and aggressive management of her cancer-related symptoms. This would best be supported with hospice care…[The patient] desires a consultation to hear about in-home hospice care at this time. We reviewed that this would allow her to have symptoms managed at home without coming in to the ED or hospital. She is already DNR/DNI
	Small cell lung cancer: Options include weekly paclitaxel or hospice. There is a low response rate to paclitaxel given his refractory disease. However his performance status is still adequate and he wants to try more chemo. I went over the risks, benefits and potential side effects with the patient in detail. The patient signed informed consent. Poor prognosis discussed with patient and wife. Likely months to live
**Insufficient documentation** Information documented is inadequate or too vague to provide insight into patient/family goals	# Goals of care – Patient previously on home hospice services + home health services – no longer on hospice and does not want to pursue this

## DISCUSSION

In this mixed-methods study, we demonstrate that a review of EHRs can reliably classify GOC for most hospitalized patients at high risk of dying into three discrete and clinically relevant categories—life extension, maintain or improve function, and comfort-focused. Second, we find that the most common trajectory of GOC over time among such patients was from life extension to comfort-focused, particularly among the roughly one-third who died within 6 months of hospitalization. Third, our qualitative analyses reveal that ambiguity expressed by patients and families regarding their GOC and insufficient documentation are the main reasons why GOC cannot be discretely classified for a minority of patients, and why inter-rater reliability was imperfect.

This study provides proof-of-concept of a conceptual framework to identify patients’ individual GOC from unstructured clinical note data in the EHR. Prior studies using myriad methods have reported rates of documented GOC discussions ranging from 0 to 93% among patients with serious illness.^9,31,32^ Our findings likely represent an improvement from others for GOC detection (had we focused only on ACP notes, for example, we would have missed 65% of GOC conversations) and the 22% of patients with no identified GOC conversation are a reasonable estimate of a “true negative” for having had no GOC conversation. While there are undoubtedly instances in which high-quality GOC conversations occurred, if they were not documented within the EHR, that remains a problem for future care providers. These findings highlight how much work is still needed to increase the conduct and documentation of GOC conversations.

Moreover, we extended the measure beyond the typical binary outcome of the presence of a documented GOC discussion^10,19,33^ to further categorize patients’ goals from the documented discussions. This approach leverages contemporaneously documented GOC expressed by patients or surrogates during a serious illness conversation with their clinicians, thus avoiding the recall and desirability biases and data missingness that plague patient-reported GOC collected independent of real-world context.^12,34^ Importantly, this work suggests the potential for scalability by training text-based classification tools (e.g., natural language processing and machine learning methods) on reliable labels provided by clinicians-in-training, with only a minority requiring content expert review. If so, this would address a major challenge to serious illness research—the absence of a pragmatic, reliable, and patient-centered measure of goal-concordant care.

Indeed, although we find that goals are unclear in approximately 1 in 7 GOC discussions, our qualitative findings suggest that this classification is often a “true negative”—i.e., the categorization of these goals as unclear captured the real uncertainty that many patients and families experience in serious illness decision-making, the potential for multiple goals to co-exist, and the logical conditionality of some patients’ goals (e.g., if function cannot improve or be maintained, then focus on comfort). Further, the majority of patients with initially unclear goals had a subsequently documented GOC discussion in which goals were clarified, reflecting the important role of time in providing clarity^35^ and the longitudinal nature of high-quality communication in serious illness. Limited documentation by clinicians also contributed to coders’ uncertainty in definitively classifying patients’ goals, which could be addressed by designing EHR note templates that prompt more informative documentation of GOC discussions. Future iterations of a GOC categorization framework should disaggregate goals that are truly unclear from those with inferior documentation.

We observed a clear pattern of goals shifting toward comfort-focused care during the 12-month follow-up. This may represent the natural history of GOC among a cohort of patients with a limited prognosis. However, it also likely reflects clinicians’ tendencies to preferentially revisit GOC discussions when patients experience disease progression or a complication,^36,37^ particularly when goals are initially unclear or focused on longevity. This is supported by our finding that decedents were more likely to have > 1 GOC discussion, and most had new comfort-focused goals documented within days prior to death. More work is needed to validate and extend our exploratory findings of GOC trajectories in a prospective cohort to identify opportunities to improve the patient-centeredness of approaches to recurrent GOC discussions.^38^

Strengths of this study include a broadly defined population of seriously ill hospitalized adults that promotes the generalizability of this promising method for classifying GOC. The four classifications of GOC are sufficiently broad to allow for the categorization of heterogeneous goals across individuals, while still representing distinct and common approaches to care delivery in serious illness. We used a comprehensive definition to identify GOC discussions in clinical notes as was done in prior studies,^5,19^ but improved the method’s patient-centeredness by excluding isolated mentions of code status or a single treatment preference (e.g., radiation) that lacked any additional context of patients’ goals, values, or overall care preferences. Finally, our comprehensive chart review resulted in a more inclusive and generalizable sample of documented GOC discussions compared to relying on EHR note types or templates specific to GOC discussions.

The study must also be considered in light of several limitations. First, our study was limited to GOC documentation within the EHR at a single health system. We cannot comment on GOC documented in external health care systems during the study period. The culture and practice of GOC communication and documentation may differ across hospitals and health systems, which could impact the generalizability of the goals classification framework evaluated in this study. Moreover, while 38% of our study population identified as Black, we had low rates of representation of individuals from other racial and ethnic minority backgrounds. External validation in another health care system and among a more diverse cohort of patients is necessary. Second, documented GOC discussions are infused with clinicians’ interpretation and summary of the goals patients or their surrogates express (though the EHR is the primary means by which such medical information is communicated between clinicians). Relatedly, this method does not capture the uniqueness of individual patients’ goals (for example, to live long enough to make it to a family member’s wedding or to be sufficiently functional to care for or entertain grandchildren). A prospective validation study would allow for contemporaneous corroboration of patients’ GOC classification with the gold standard of patient- or surrogate-reported goals. Third, we focused on hospitalized patients with a limited prognosis based on expert recommendations regarding timely GOC discussions and to ensure a sufficient sample of documented GOC discussions to review. This study design choice undoubtedly influenced the epidemiology of the GOC discussions we report. For example, we may underestimate the proportion of patients at high mortality risk with comfort-focused goals at baseline because avoiding hospitalization is commonly desired by such patients. Similarly, a less sick cohort would likely have revealed fewer GOC discussions per patient. Fourth, while 110 GOC (33% of the total sample) were re-reviewed through the adjudication process and qualitative assessment of unclear goals, we did not re-review the 228 goals that had clear and concordant classification. As such, we cannot be certain that coder drift did not occur. Finally, any efforts to operationalize categorizing GOC at scale will need to advance automated techniques as the current approach of dual-chart review is resource-intensive.

## CONCLUSIONS

In conclusion, classifying seriously ill patients’ goals into distinct and clinically relevant categories using GOC discussions documented in the EHR is feasible. This GOC classification framework enhances the patient-centeredness of documented GOC discussion as an outcome measure, applies across different serious illnesses and throughout the duration of disease, and advances the much-needed development of a pragmatic approach to measuring goal-concordant care. Future work is needed to externally validate, optimize, and ideally automate this approach.

### Supplementary Information

Below is the link to the electronic supplementary material.

Supplementary file1 (DOCX 163 KB)

## Data Availability

Deidentified participant data can be made available to researchers after publication and with approval of a methodologically sound proposal (catherine.auriemma@pennmedicine.upenn.edu).
