# Supplementary material for: Classification of Documented Goals of Care Among Hospitalized Patients with High Mortality Risk: a Mixed-Methods Feasibility Study
Source: J Gen Intern Med. 2024 May 6;39(10):1839–49. doi: 10.1007/s11606-024-08773-z (PMC11282019; doi:10.1007/s11606-024-08773-z)
Supplement: Supplementary file 1 — Supplementary file1 (DOCX 163 KB) [file 11606_2024_8773_MOESM1_ESM.docx]

**Supplemental Table 1.** Patient characteristics

| **Clinical Variable^*^** | **All patients**  **(n = 109)** |
| --- | --- |
| Age, years | 70 (63, 79) |
| Female sex, % | 53 (49) |
| Race |  |
| White, % | 63 (58) |
| Black, % | 41 (38) |
| Multi-racial, % | 2 (2) |
| Asian, % | 1 (1) |
| Other, % | 2 (2) |
| Ethnicity |  |
| Not Hispanic/Latinx, % | 108 (98) |
| Prefer not to answer, % | 1 (2) |
| Religion, % |  |
| Christian | 40 (37) |
| Roman Catholic | 31 (28) |
| Jewish | 13 (12) |
| Other | 5 (5) |
| Unknown | 20 (18) |
| Primary language |  |
| English | 106 (97) |
| Other | 3 (3) |
| Insurance |  |
| Medicare | 83 (76) |
| Private | 22 (20) |
| Medicaid | 4 (4) |
| Chronic illness category**, % |  |
| Cardiac, % | 76 (70) |
| Metastatic cancer, % | 50 (45) |
| Renal, % | 42 (39) |
| Pulmonary, % | 23 (21) |
| Neurologic, % | 22 (20) |
| Liver, % | 16 (15) |
| Lymphoma, % | 13 (12) |
| Solid tumor without metastases, % | 10 (9) |
| Elixhauser score | 6 (4, 8) |
| Predicted six-month mortality |  |
| High 0.5-0.74 | 54 (50) |
| Very high 0.75-1 | 55 (50) |
| Admit type |  |
| Medical | 85 (82) |
| Surgical | 19 (18) |
| Admission source |  |
| Emergency department | 85 (78) |
| Direct from clinic or home | 7 (6) |
| Another institution | 17 (16) |
| Enrollment hospitalization LOS | 7.8 (4.8, 12.0) |
| Palliative care consultation during enrollment hospitalization | 43 (39) |
| ICU stay during enrollment hospitalization | 35 (32) |
| Palliative care consultation during follow-up | 14 (15) |
| Rehospitalization during follow-up | 54 (58) |
| Home care enrollment during follow-up | 61 (66) |
| * Data shown as mean + standard deviation, number (percent), or median (interquartile range) as appropriate  ** Disease categories determined based on ICD-10 codes for Elixhauser comorbidity scores | |

**Supplemental Table 2.** Distribution of electronic health record note types containing documented goals-of-care discussions (n=338).

| Note Type | Frequency (%) |
| --- | --- |
| Advance care planning | 119 (35.2) |
| Progress (inpatient) | 85 (25.1) |
| Consult/Consultation | 47 (13.9) |
| Progress (outpatient) | 32 (9.5) |
| Home care visit | 21 (6.2) |
| History and physical | 19 (5.6) |
| Discharge summary | 5 (1.5) |
| Emergency department provider | 5 (1.5) |
| Case management and social work | 3 (0.9) |
| Pastoral care | 1 (0.3) |
| Significant event | 1 (0.3) |
